# Case report: Endoscopic endonasal transposterior clinoid approach for resection of posterior clinoid process meningioma: technical notes and literature review

**DOI:** 10.3389/fonc.2024.1368277

**Published:** 2024-06-11

**Authors:** Steven Awyono, Kazuhito Takeuchi, Eiji Ito, Yuichi Nagata, Nyoman Golden, Tjokorda Gde Bagus Mahadewa, Ryuta Saito

**Affiliations:** ^1^ Neurosurgery Division, Department of Surgery, Faculty of Medicine, Udayana University, Prof. Dr. I.G.N.G. Ngoerah General Hospital, Bali, Indonesia; ^2^ Department of Neurosurgery, Nagoya University Graduate School of Medicine, Nagoya, Japan

**Keywords:** endoscopic endonasal, posterior clinoid process meningioma, posterior clinoidectomy, technical notes, transposterior clinoid approach

## Abstract

**Background:**

Posterior clinoid process (PCP) meningioma is an exceedingly rare entity. It remains the most challenging skull base lesion for neurosurgeons due to its treacherous location that insinuates amongst critical neurovascular structures. This article will describe the technical notes using the endoscopic endonasal approach that provide the earliest devascularization and detachment of the tumor PCP meningioma.

**Methods:**

We are introducing the surgical implementation of an endoscopic endonasal approach to removing PCP meningioma. Furthermore, we perform a literature review of posterior clinoid process meningioma that undergoes surgical intervention, then summarize the benefits and limitations of each approach.

**Results:**

We present a case of right PCP meningioma that was removed using an endoscopic endonasal approach through the transposterior clinoid corridor in a 52-year-old-woman. We describe the technical notes in performing this approach to have the earliest devascularization and detachment of the tumor by performing posterior clinoidectomy. Safe tumor removal is performed with a wide and clear view of the surrounding neurovascular structure. Based on our database search, we found nine articles reported on the surgical management of PCP meningiomas, with a total number of 15 cases. All of the reported cases performed the tumor removal using the transcranial approach.

**Conclusion:**

The endoscopic endonasal transposterior clinoid approach circumvents all disadvantages faced by the traditional transcranial approach, providing the earliest approach to devascularized and detaching the tumor from its attachment at PCP. This approach demonstrates safety and efficacy, making it an acceptable alternative for PCP meningioma resections.

## Introduction

Posterior clinoid process (PCP) meningioma is exceedingly rare entity and remains the most challenging skull base lesion for neurosurgeons (1–8). Clinical manifestation and radiological findings are rarely discussed and reported because of their dearness with terminological puzzlement (3, 4). The strategy for surgical intervention remains controversial due to its proximity to critical neurovascular structures susceptible to injury. PCP is in the depth of the skull base that forces the neurosurgeon to work in a very limited area between the optic nerve, oculomotor nerve, pituitary stalk, internal carotid artery (ICA), and its perforators (1–5, 8). The basic concept of this surgery is early devascularization (1, 9). Numerous surgical approaches, from microscopic transcranial to endoscopic assisted transcranial surgery already been described for approaching the PCP to minimize surgical morbidity and expand the surgical resection with its own consequences. However, the most optimal approach is still under debate because these approaches have their drawbacks (1–5, 8–11).

Hereby, we present a case of PCP meningioma in a 52-year-old woman that was removed using an endoscopic endonasal approach (EEA) through the transposterior clinoid corridor by underlining its technical notes and surgical nuances. We provide a detailed description of the clinical history, examination, operative technique.

## Methods

One patient with PCP meningioma who underwent EEA through the transposterior clinoid corridor was reported in this study. Consent was obtained from the patient who were fully informed of the risks of the surgery. The clinical presentation, imaging, operative technique, and postoperative condition were detailed and described.

We searched PubMed to locate articles that documented the surgical approach used to treat PCP meningioma. The search was performed using the keywords posterior clinoid process, meningioma, and surgery. The inclusion criteria were defined as patients who were undergoing surgical treatment for PCP meningiomas, with the requirement that the surgical procedure be described and written in English.

We provide a concise overview of the surgical methods outlined in existing literature, thoroughly examine each surgical procedure used for PCP meningioma that has been previously documented and evaluate the merits and disadvantages of each approach in terms of surgical outcomes.

## Results

### Case illustration

A 52-year-old woman was brought to the outpatient clinic complaining about headaches. This symptom has been progressively worsening for approximately the last two months. There is no history of seizures, nausea or vomiting. Both ophthalmological and neurological examinations are within normal limits.

**Figure 1:**
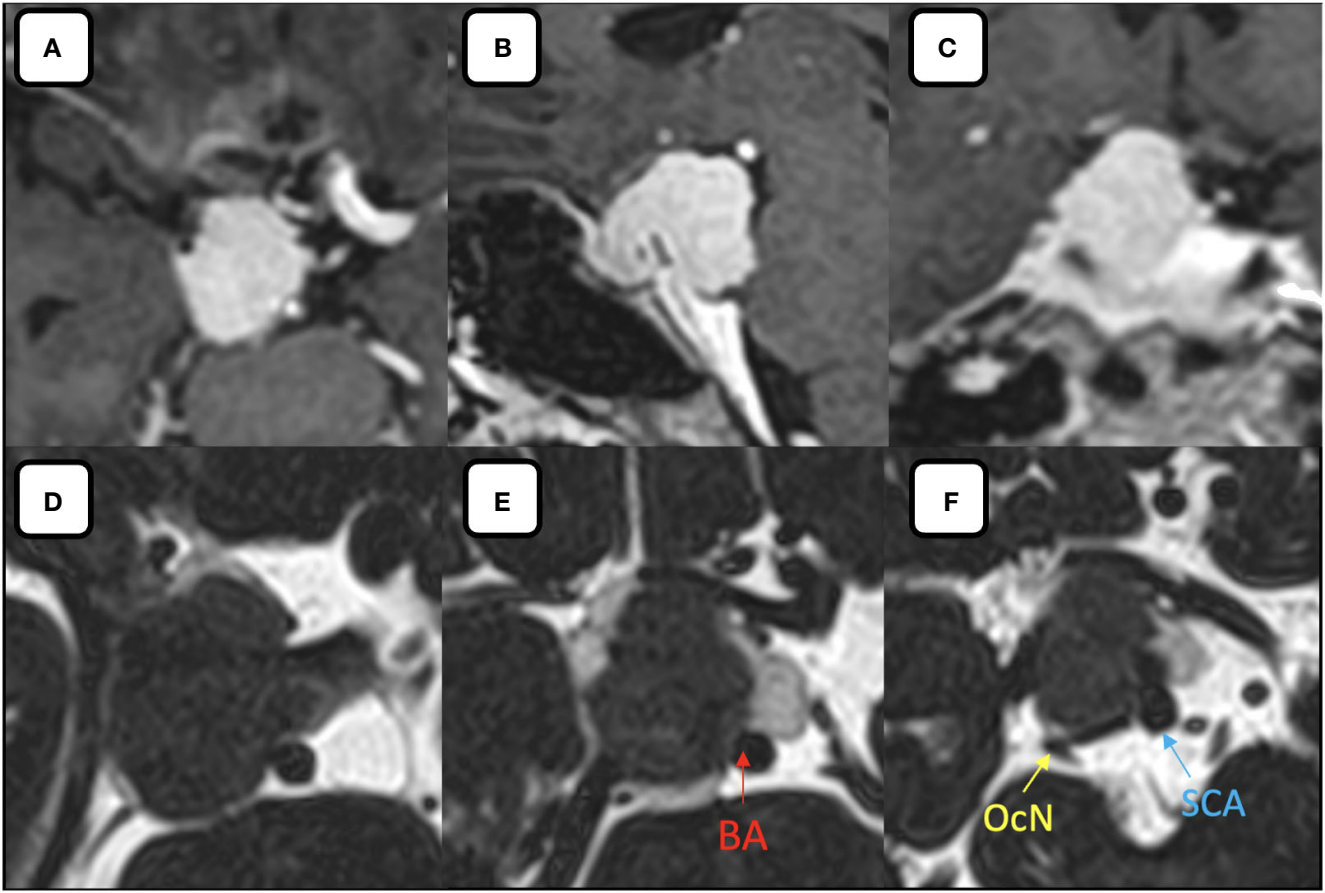
**(A–C)** Post contrast **(A)** axial, **(B)** sagittal, **(C)** coronal MR images showing the homogenous enhancement of the lesion relative to its surrounding structures. **(D–F)** Axial MR-Cisternography denotes the anterior displacement of the ICA, partial encasement of the basilar artery by the tumor, postero-lateral displaced oculomotor nerve and superior cerebellar artery by the tumor. BA, basilar artery; OcN, oculomotor nerve; SCA, superior cerebellar artery.

Magnetic resonance (MR) imaging revealed an extra-axial mass that was hypointense on T1-weighted, with homogenous contrast enhancement with cerebrospinal fluid (CSF) cleft sign around the lesion. This lesion is centered over the right PCP with a maximum diameter of 25 mm (**Figure 1**) suggested as PCP meningioma with mild brainstem compression. MR angiography showed that the ICA had pushed anteriorly, with the posterior communicating artery pushed to the infero-lateral portion. After discussing with the patient and family, we planned to perform an EEA for her tumor through the transposterior clinoid corridor.

### Operative procedure

**Figure 2:**
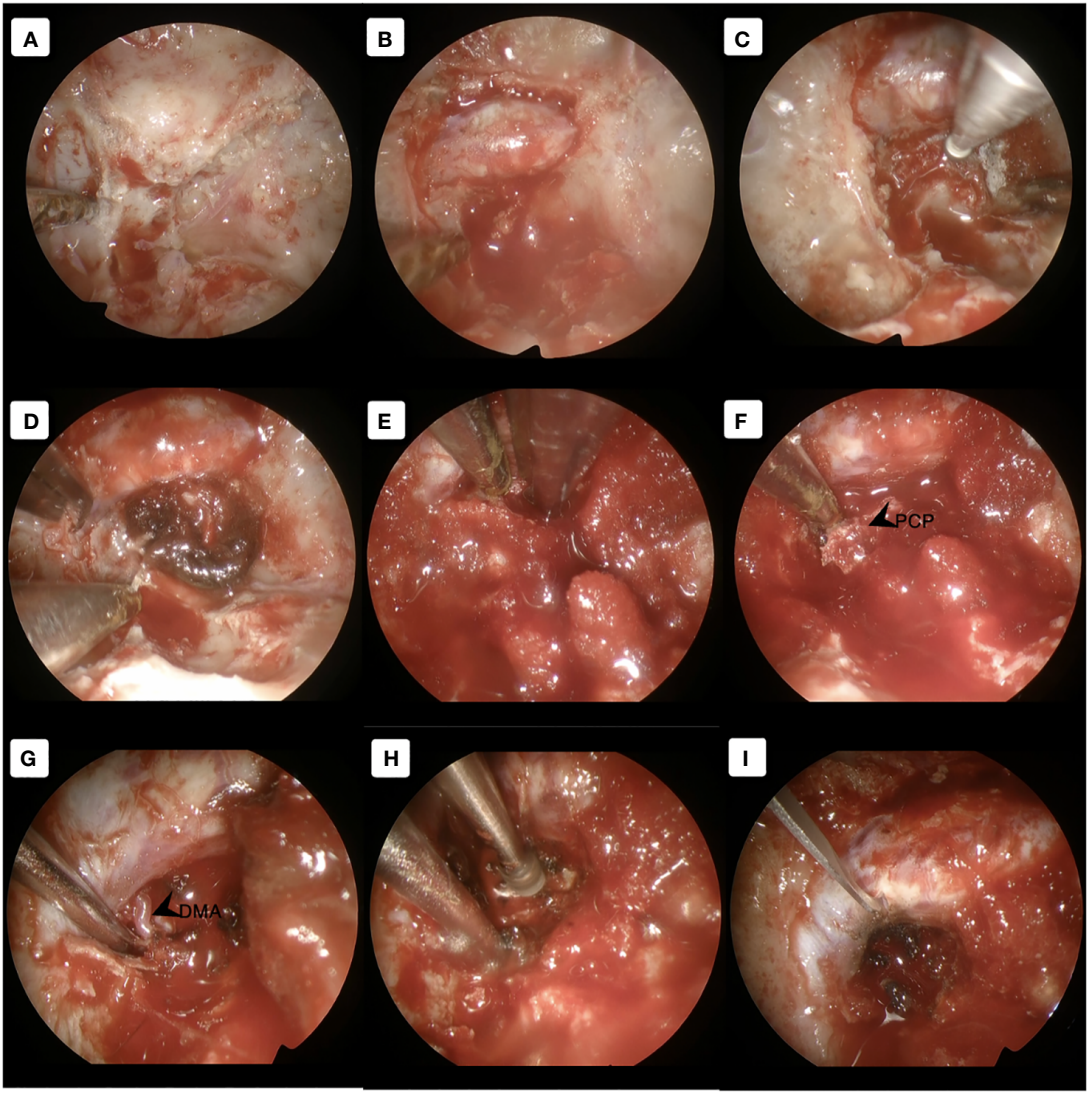
Approach the PCP using the transseptal approach. **(A)** Exposing the skull base and identifying the dorsum sella, clival and carotid canal **(B)** Opening the dorsum sella and **(C)** drilling out until paper-thin appearance. **(D)** Deskeletonized the carotid canal as the lateral border of the PCP. **(E)** Removed the dorsum sellae using the Kerrison rongeur to detach the PCP completely. **(F)** Shifting the PCP towards the midline to complete the posterior clinoidectomy. **(G)** Visualization of the main feeder by the dorsal meningeal artery. **(H)** The ossified dura was drilled out to expose the tumor. **(I)** Dural opening until the edge of the bone flap. DMA, dorsal meningeal artery; PCP, posterior clinoid process.

**Figure 3:**
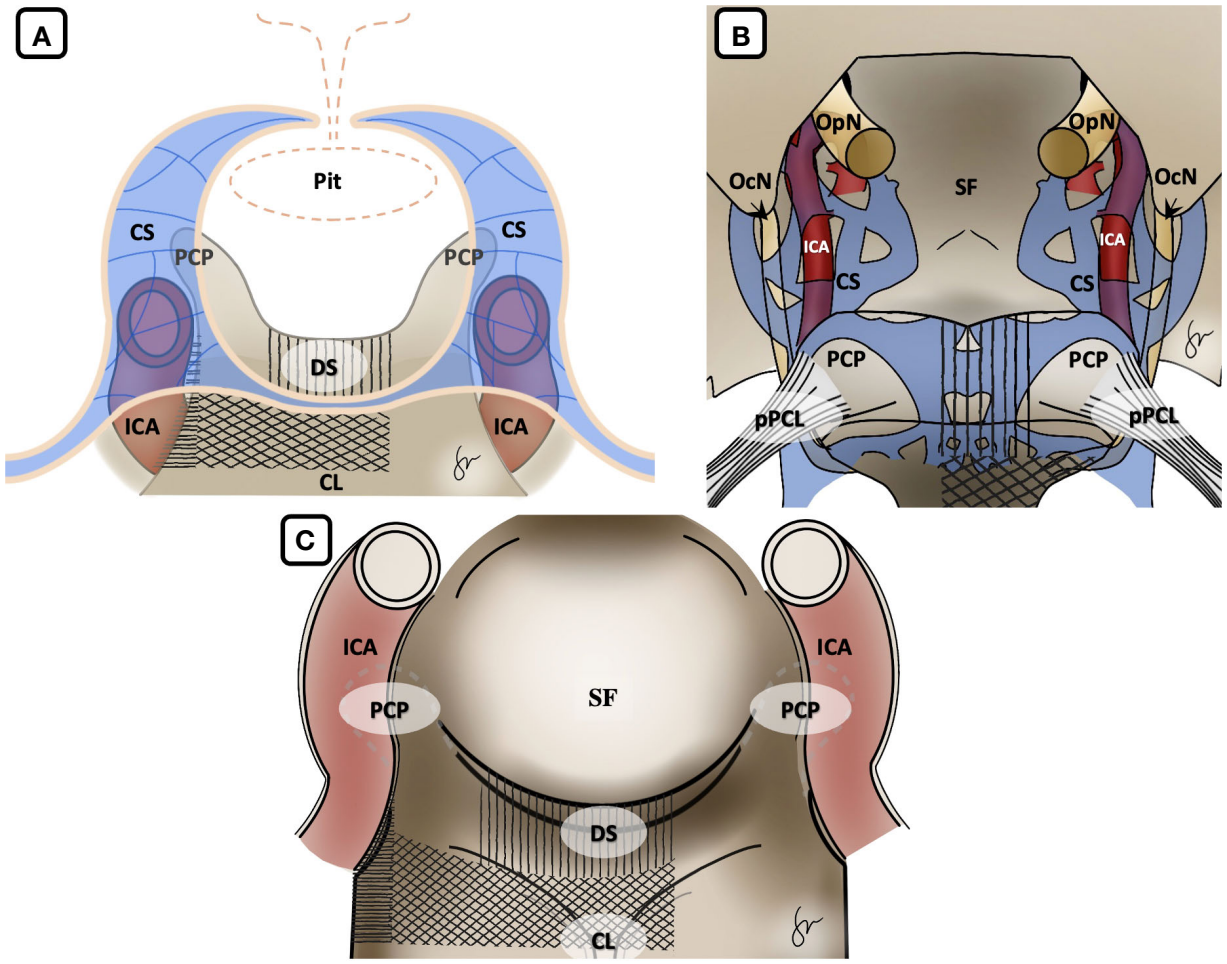
Illustration of Posterior Clinoidectomy by disconnection of the surrounding bony structures. **(A)** Coronal view, **(B)** axial view from above and **(C)** endoscopic view. It is important to acknowledge that in the initial stage, the dorsum sella was removed, followed by the excision of the upper clival bone. Finally, the medial carotid canal was removed by using the drill and Kerrison rongeur. Vertical hashed lines: dorsum sella drilling; Diagonal hashed lines: upper clival drilling; Horizontal hashed lines: carotid canal drilling. CL, clival; CS, cavernous sinus; DS, dorsum sellae; ICA, internal carotid artery; OcN, oculomotor nerve; OpN, optic nerve; PCP, posterior clinoid process; Pit, pituitary gland; pPCL, posterior petroclinoidal ligament; SF, sellar floor.

**Figure 4:**
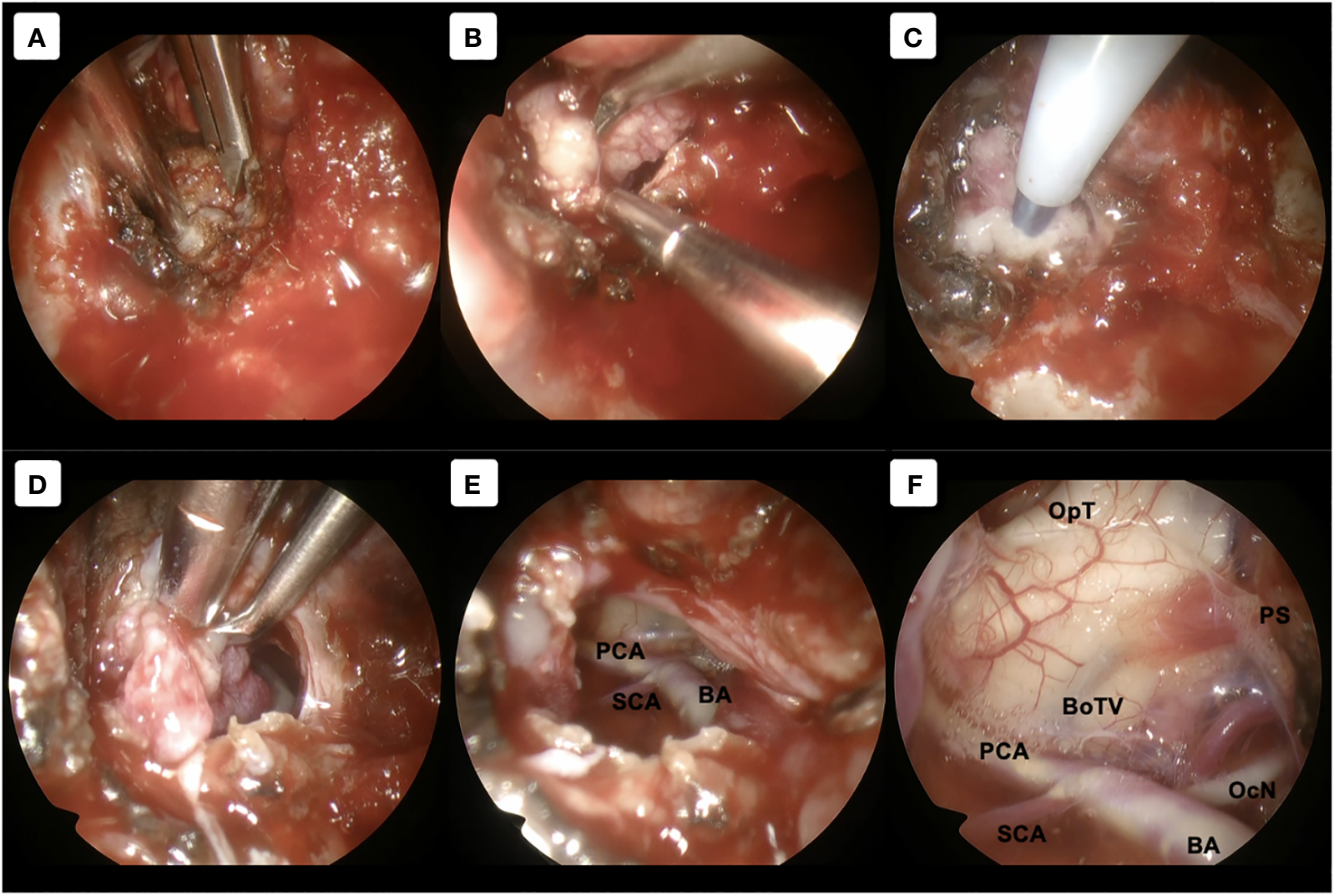
Tumor removal procedure. **(A–C)** Using 30-degree angled endoscope. **(A)** Direct exposure to the tumor without any obstruction by the surrounding structures was achieved. **(B, C)** Further tumor debulking was performed using curettage and sonographic aspirator Sonomet. **(D–F)** Using 70-degree angled endoscope. **(D)** Unrestricted mobilization of the tumor, resulting from its prompt detachment from the PCP, enhances our dissection procedure to assess the adjacent anatomical structures. **(E)** Tumor can be dissected from the surroundings with direct and clear visualization. **(F)** Evaluation of the surrounding structures after gross total removal. BA, basilar artery; BoTV, base of third ventricle; OcN, oculomotor nerve; OpT, optic tract; PCA, posterior cerebral artery; PS, pituitary stalk; SCA, superior cerebellar artery.

After induction of general anesthesia, the patient was positioned supine. A right-sided uninostril approach was selected. We used an endoscopic-transseptal approach to expose the sphenoid sinus (Video 1). A sphenoidectomy was performed to expose the sellar floor down to the upper-mid clival, which is essential for a successful posterior clinoidectomy. This exposure facilitates the identification and allows access to the PCP (**Figures 2A, B**). A technique was employed to mobilize the PCP en-bloc from the clival dura medially and its ligamentous connections on its anterior and posterior sides. The PCP is first detached from the dorsum sellae using a high-speed drill until it displays a surface texture mimicking a paper-thin appearance (**Figure 2C**). Next, we skeletonized the carotid canal that connects to the PCP (**Figure 2D**). This preparation facilitates the simpler resection of the bone flap while minimizing the risk of causing harm to the underlying tissue. Care must be taken as the dural lining of the posterior lobe is thinner than that of the anterior, making it more sensitive and susceptible to manipulation and injury (12). The Kerrison rongeur was then introduced to remove the dorsum sellae completely (**Figure 2E**). Following the separation of the PCP from the dorsum sella, the dura medial to the PCP is dissected using a blunt dissector. It is important to notice that PCP has superior projection at its apex and posterolateral projection that attaches to the petrous bone via posterior petroclinoid ligament. This posterolateral projection is the most adherent portion of PCP, and its complete separation is required in order to detach the PCP completely. These procedures allow the shifting of the PCP towards the midline to remove the PCP completely (**Figure 2F**). After performing posterior clinoidectomy, we then faced venous bleeding from the cavernous sinus laterally, which was then controlled by using Surgiflo^®^ (Ethicon, Inc). The dorsal meningeal artery was identified from the inferior surface as the main arterial feeder and coagulated without any difficulty (**Figure 2G**). We opened the dura until the edge of the bone flap, and the dura attached to the tumor was calcified and drilled out to expose the tumor (**Figures 2H, I**). The anatomical and technical procedure was illustrated (**Figure 3**). Intraoperatively, the tumor was grey-reddish in color, with firm consistency and moderately avascular due to early devascularization (**Figure 4A**). The tumor was debulked comfortably using curettage and Sonopet^®^ (Stryker, Inc) ultrasonic surgical aspirator without any significant bleeding (**Figures 4B, C**). The debulking process was simultaneously performed with tumor dissection by evaluating the arachnoidal plane around the tumor. The tumor is then freely mobilized due to early detachment from its base on the PCP, which facilitates our dissection process to evaluate the surrounding anatomical structures (**Figure 4D**). During the dissection of the tumor, we identify the ICA with its main branches and oculomotor nerve that runs on the lateral side of the tumor. Pituitary stalk was observed on the superomedial direction and basilar artery posteriorly (**Figures 4E, F**).

**Figure 5:**
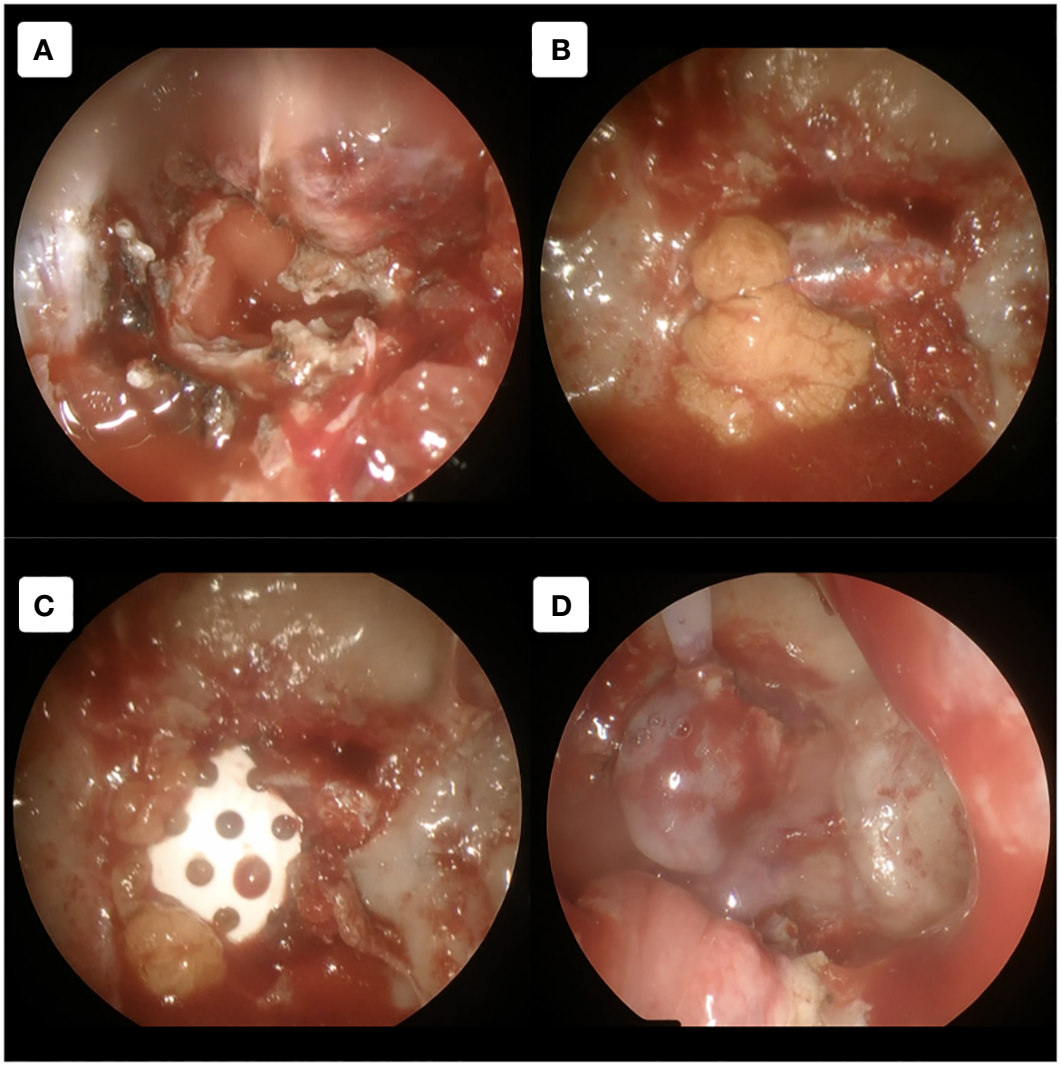
Multilayer dural closure technique. **(A)** Non-autologous dural substitute agent was used as an inlay layer. **(B)** Then abdominal fat graft was used to cover it with additional dural suturing to ensure the graft remained in place. **(C)** Bony defect reconstruction was done using Superfixorb (absorbable bone graft). **(D)** The final layer of mucosal flap was used to cover all the defects with additional fibrin glue.

Hemostasis was achieved by using Surgicel^®^ (Ethicon, Inc), and saline irrigation. Multilayer dural closure was performed to prevent postoperative CSF, first by using Duragen^®^ (Integra, Inc) under the dura as an “inlay” fashion (**Figure 5A**) covered by harvested abdominal fat, then suturing the dura to compress the multilayer closure (**Figure 5B**) to prevent CSF leakage. We used Superfixorb^®^ (Muranaka, Inc) as a solid component to cover the entire skull base defect (**Figure 5C**). The mucosal flap serves as the final layer, and fibrin glue is applied to it (**Figure 5D**).

### Postoperative management

**Figure 6:**
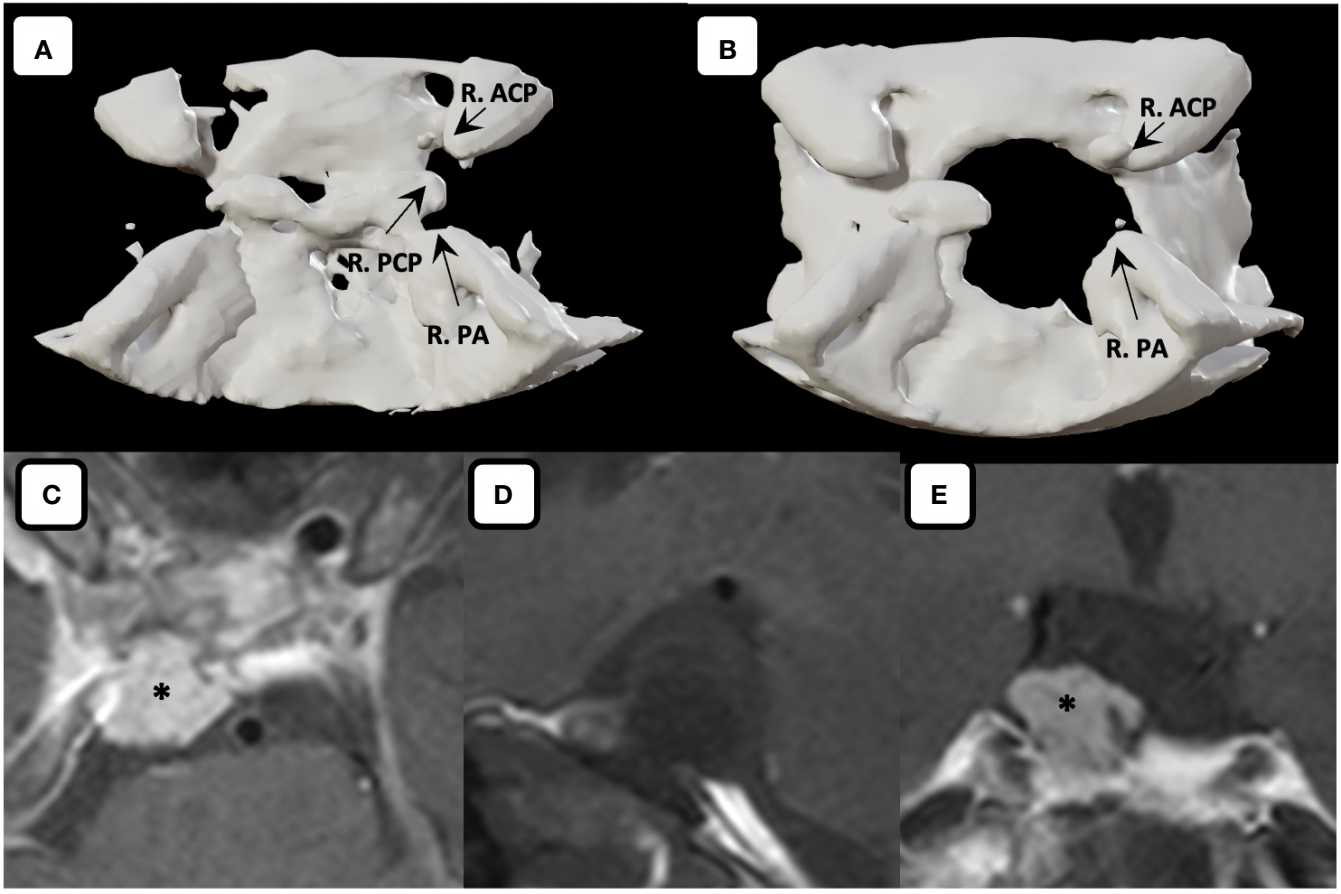
Three-dimensional computed tomography. Comparison between **(A)** preoperative and **(B)** postoperative three-dimensional computed tomography showed the skull base defect after performing right posterior clinoidectomy. Postoperative MR imaging of the tumor. **(C)** Axial, **(D)** sagittal (fat suppressed imaging), **(E)** coronal MR images showing gross total tumor removal are achieved with the fat graft covering the defects on the skull base without any sign of leakage. Asterisk denotes the fat graft on MR imaging. R. ACP, right anterior clinoid process; R. PA, right petrous apex; R. PCP, right posterior clinoid process.

Postoperative courses were uneventful. On 3^rd^ month follow-up, the patient has not any neurological deficit. Histopathological examination showed findings consistent with transitional meningioma, WHO grade I. Postoperative computed tomography scan showed right posterior clinoidectomy with skull base reconstruction using the analogous bone graft (**Figures 6A, B**). MR imaging revealed gross total removal of the tumor without any CSF leakage (**Figures 6C-E**).

### Literature review

We found nine articles reported on the surgical management for PCP meningiomas, with the total number of 15 cases (**Table 1**) (1–5, 7, 8, 10, 11). All of the surgery operated through transcranial approach, and one of it used an endoscopic-assisted far lateral supracerebellar infratentorial approach. The gross total removal was 5/15 (37.5%) cases, with near total removal and subtotal removal of 4/15 (22.5%) and 6/15 (40%), respectively. Several transcranial approaches used for resection of PCP meningioma are transcavernous-transellar, sigmoid-trans petrosal, tranzygomatic subtemporal, basal frontotemporal orbital-zygomatic transsylvian, frontotemporal-transsylvian, retrosigmoid, combined transpetrosal and endoscopic-assisted far lateral supracerebellar infratentorial approach. Several complications reported previously are hemiparesis 4/15 (22.5%), cranial nerve palsy 4/15(22.5%), brain infarction 2/15 (11.3%), and hemianopsia 1/15 (5.6%).

**Table 1:** Summarized of published report describing the surgical removal of posterior clinoid meningioma.

Authors	Age	Sex	Max D (mm)	Symptoms	Approach	Extent of tumor removal	Complications
Goto et al. n= 5 (1)	45 to 58 years; Mean of Zage = 51.6	M= 3 F= 2	N/D	• N/D	• Presignoimdal transpetrosal approach (n=4) • Combined transpetrosal and OZ approach (n=1)	• Gross total (n=3) • Near total (n=1) • Partial (n=1)	• None
Shukla et al. n= 2 (2)	50	F	40 mm^1^	• Headache • Diplopia (oculomotor nerve palsy)	• Transzygomatic subtemporal	• Total	• None
	41	F	45 mm^1^	• Headache • Diplopia (oculomotor nerve palsy)	• Transzygomatic subtemporal	• Partial	• Hemiparesis • Hemianopsia • Partial Oculomotor nerve paresis • Infarction in the territory of AChoA
Sodhi et al. n=1 (3)	48	F	30^!^	• Headache • Visual deficit • Left homonymous hemianopia	• FTOZ	• Near total	• Transient ptosis
Takase et al. n= 2 (4)	54	F	20	• Left Opthalmoplegia • Visual loss	• FT	• Near total	• None
	62	F	65	• Akinesia • Amnesia • Visual deficit • Homonymous hemianopia	• FTOZ	• Subtotal	• Transient hemiparesis • Infarction in the territory of LSA
Nanda et al. n= 1 (5)	66	F	45^1^	• Facial Pain	• Retrosigmoid	• Subtotal	• Transient hemiparesis
Young et al. n= 1 (11)	53	F	N/D	• N/D	• Orbitozygomatic transpetrosal	• Near total	• Mild Oculomotor and trigeminal nerve palsy
Ohba et al. n= 1 (10)	60	M	45^1^	• Diplopia • Right ptosis	• Tranzsygomatic	• Subtotal	• Left hemiparesis (brainstem infarction)
Wu et al. n= 1 (6)	68	F	50^1^	• Hemiparesis • Seizure	• Combined transpetrosal	• Subtotal	• Temporary abducens nerve palsy
Bai et al. n= 1 (8)	67	F	25^1^	• Visual deficit	• Endoscopic far-lateral supracerebellar infratentorial	• Total	• None
Present case n= 1	54	F	40	• Visual deficit	• Endoscopic endonasal transposterior clinoid	• Total	• None

AChoA, anterior choroidal artery; FT, frontotemporal; FTOZ, frontotemporal orbitozygomatic; LSA, lenticulostriate artery; N/D, not described; OZ, orbitozygomatic.

^1^radiologically expected size.

## Discussion

### Terminology and incidence

Several terminologies have been implemented to describe tumors around the dorsum sellae to the ventral of brainstem area, which includes dorsum sellae or clival meningioma, retrosellar meningioma and PCP meningioma (2, 9, 13, 14). These tumors were later classified by Sodhi et al. into 2 types based on anatomical consideration of the tumor attachment (3).

- Type 1 - meningioma dorsum sella or upper clival if the tumor is located between the two PCPs with the tumor attachment on the dorsum sella or upper clival region.
- Type 2 – PCP meningioma if the tumor surrounds the PCP with the attachment of the tumor located on the PCP.

Due to the lack of information regarding the characteristics and surgical approaches regarding PCP meningioma, we try to summarize several surgical approaches that have been implemented previously and present new nuances regarding endonasal endoscopic surgery techniques to treat PCP meningioma.

PCP is located centrally within the skull base, corresponding to the superolateral part of the dorsum sella (8). It is connected to the ACP anteriorly through the interclinoid ligament, which may be ossified in some patients and considered an anatomical variant (15, 16). PCP is also surrounded by many critical vascular structures, including the basilar artery posteriorly and ICA together with its perforators in the antero-lateral projection. The ascending segment of the ICA is located on the posterior aspect of the cavernous sinus, whereas the posterior bend of the ICA is situated on the lateral side of the PCP (15, 17).

The optic chiasm and the optic tract are located on the antero-superior projection of the PCP (4, 8, 15, 17). The oculomotor nerve that gives entrance to the cavernous sinus is located just antero-lateral to the PCP so-called oculomotor trigone that easily compressed by the tumor (1, 3, 4, 8, 15, 17).

### Surgical approaches for PCP meningioma

PCP meningioma is exceedingly rare and enormously challenging entities. The treacherous anatomical location of PCP poses a challenge to skull base neurosurgeons for the approach to this entity (4, 5, 8). The basic concept of PCP meningioma surgery is to perform early devascularization with meticulous dissection of the tumor from surrounding neurovascular structures (1, 4, 8) Several surgical approaches have been implemented to manage PCP meningioma with various benefits and drawbacks, including frontotemporal, basal frontotemporal orbito-zygomatic, transsellar transcavernous, transzygomatic subtemporal, presigmoid transpetrosal with petrosectomy modifications, retrosigmoid, and endoscopic assisted supracerebellar infratentorial (**Table 2**) (1–5, 8, 10, 11).

**Table 2:** Summary of implemented surgical approach of posterior clinoid process meningioma.

Year	Approach	Benefits	Drawbacks	Key points
**2003**	**Transcavernous-transsellar approach** Dolenc, 2003 (12)	• Direct exposure to the ICA and its branches facilitate tumor dissection, especially from the anterior choroidal artery	• Anterior displacement of the ICA that hindered direct tumor visualization • Peeling off the cavernous sinus dura that may lead to cranial nerves deficit and sinus thrombosis as PCP meningioma may obstruct the inferior petrosal sinus • Injury of ICA perforators while performing devascularization of the tumor	Extradural bone work must be form initiallya (unroofing the orbit; removing the sphenoid wing, anterior clinoid process and the wall of optic canal; splitting the dura at superior orbital fissure.
**2009**	**Presigmoid, transpetrosal approach** Goto & Ohata, 2009 (1)	• Removal of the infratentorial tumor (anterior petrosectomy) • Exposure to the proximal oculomotor nerve to prevent injury • Allows direct dissection of the ICA, which displaced to the antero-superior projection • Provides direct visualization to the optic chiasm	• Complex surgery • Have a risk of hearing loss with facial nerve injury while drilling the bone	• Anterior petrosectomy is mandatory for tumor that extends to infratentorial region • Meticulous dural elevation from the middle cranial base to preserve venous return • Early identification of proximal oculomotor nerve
**2010**	**Transzygomatic Subtemporal approach** Ohba et al., 2010 (10) Shukla et al., 2012 (2)	• Early devascularization • Direct exposure of the PCP • Wide exposure to upper clival by splitting the tentorium	• Limited by anterior extension of the tumor through the prechiasmatic cistern • Difficulty to decompress the optic canal • Temporal lobe retraction may lead to contusion • Injury to the vein of Labbe; prolonged vein compression	• Brain relaxation is mandatory to minimize surgical complications, which may be aided by placing a lumbar drain preoperatively
**2015**	**Basal FTOZ-transylvian approach** Sodhi et al., 2015 (3) Takase et al., 2016 (4)	• Allow access to the PCP • Minimal temporal lobe retraction • Early optic canal decompression	• Incapacious surgical between critical neurovascular structures provoke an increased risk of injury to these structures	• In case of large tumor, preoperative lumbar drain may be needed to minimize surgical complications • Maybe combine with pregsimoid transpetrosal approach
**2016**	**Frontotemporal-transsylvian approach** Takase et al., 2016 (4)	• Early identification of ICA and its branches • Early decompression of the optic nerve	• Incapacious surgical between critical neurovascular structure provoke an increased risk of injury to these structures • More anterior temporal lobe retraction • Difficulty in identifying perforators which pushed anteriorly by the tumor	• Wide exposure provided by this approach by splitting the sylvian fissure
**2017**	**Retrosigmoid approach** Nanda et al., 2017 (5)	• Direct visualization while dissecting a large mass that compresses brainstem • Extent of visualization around the PCP region	• Narrow surgical corridor • Great depth, which leads to a higher risk of neurovascular injury during tumor dissection • Difficulty in performing early devascularization from the tumor base around PCP • Difficulty in visualizing anterior part of the tumor	• Two stages of surgery with frontotemporal or FTOZ may be needed to remove the tumor completely • Carefully preserved SCA and AICA • Tactful handling while dissecting tumor adherent to the brainstem • Intraoperative neuromonitoring may be helpful
**2022**	**Combined transpetrosal approach** Wu et al., 2022 (7)	• Facilitates wide exposure reaching the entire central skull base • Provide safe visualization of the brain stem • May reach the contralateral extension of the tumor	• Complex surgery • Risk of CSF leakage • Limited by anterior extension of the tumor • Difficulties when decompression of the optic canal • Tumor attachment might be visualized after sufficient tumor removal	• Provide direct exposure to the media-posterior skull base region but very invasive approach
**2022**	**EF-SCITA** Bai et al., 2022 (8)	• Longer working distance • Allow tracing of the natural course of the oculomotor nerve and trochlear nerve to prevent nerve injury • Direct exposure to the branches of ICA (including PCA and AChoA), optic chiasm and pituitary stalk • Reduce neurovascular manipulation compared to the retrosigmoid approach • Prevent vein of Labbe injury • May utilize angled endoscope, which allow visualization of anatomical structures out of the telescope axis that minimizes tumor remnants in large PCP meningiomas	• Difficulties when handling deep bleeding • Need a steep learning curve • Endanger brainstem and cerebellum • Injury to the venous drainage • Devascularization could only be achieved after tumor removal that fully exposed the PCP • Difficulty in exposing suprasellar extension of the tumor	• This approach require disconnection between tentorium, transverse sinus and tentorial incisure to visualize the neural structure around the lesion
**2023**	**Endoscopic endonasal transposterior clinoid approach** (Present case)	• Provide the earliest manner to devascularized the tumor • Directly exposed the base of the tumor • Facilitate early tumor mobilization to dissect from surrounding neurovascular structures • Wide exposure around the PCP for visualization • Minimal risk of injuring surrounding neurovascular structures	• Requires a steep learning curve • Limited by lateral extension of the tumor over the lateral border of the ICA • Risk of CSF leakage	• Posterior clinoidectomy is conducted by releasing PCP to the adjacent bony framework and ligaments • Multilayer dural closure is essential to prevent CSF leakage

AICA, anterior inferior cerebellar artery; CSF, cerebrospinal fluid; EF-SCITA, endoscopic far-lateral supracerebellar infratentorial appraoch; FTOZ, frontotemporal Orbitozygomatic; ICA, Internal Carotid Artery; PCP, posterior clinoid process; SCA, superior cerebellar artery.

Dolenc first described transcranial removal for PCP meningiomas using a transsellar transcavernous approach. Several modifications were added to this approach using a simple frontotemporal approach or additional basal extension with frontotemporal orbito-zygomatic approach. These approaches provide optic canal decompression early in the surgery and direct access to the tumor, thus facilitating tumor dissection from neurovascular structures around the tumor (1, 3, 4, 8, 13).

Concerning these approaches, direct tumor manipulation was hindered by the neurovascular structures with an incapacious surgical corridor between these structures that increased the risk of injury. Furthermore, peeling off the cavernous sinus dura may lead to cranial nerve deficit and sinus thrombosis, as PCP meningioma may obstruct the inferior petrosal sinus (2, 3, 8, 13).

Goto et al. documented their experience performing surgical resections on five cases with PCP meningioma via the presigmoid transpetrosal approach (1). This approach offers the early identification of feeding arteries and proximal oculomotor nerve that may allow us to trace it towards the involved part, potentially reducing the likelihood of nerve injury. Tumors extending to the infratentorial region may be visualized by anterior petrosectomy (1, 2, 8). Several technical issues are correlated with this approach. Therefore, preoperative evaluation is essential to achieve the maximal benefits of this approach while minimizing its complications. Evaluation of the preoperative imaging by using either CT scan or MRI to measure the transverse sigmoid junction and the angle of the petrous and clival, evaluate the pneumatization of mastoid and petrous apex, bone dehiscence over the petrous ICA, and identification of cranial nerves. These measurements also relate to patient head positioning to provide more expansive and superficial working space while drilling the bone. By using this approach with good preoperative measurement, the surgeon can comfortably use bimanual dissection of the tumor. Furthermore, the risk of CSF leakage in this approach might be prevented using a fat graft over the dural opening (1, 18, 19). Despite all the benefits, this approach is very complex and requires excessive bone drilling that might result in facial nerve dysfunction, hearing impairments, and CSF leakage (1, 2, 8). As mentioned before, these issues might be overwhelmed by proper preoperative evaluation for bone drilling to gain a wider surgical corridor, and specifically for CSF leakage, surgeons use autologous fat grafts to prevent this complication (1, 2, 8).

Several reports mentioned using the subtemporal approach for PCP meningioma. This approach provides similar benefits to the presigmoid transpetrosal approach. Additional tentorium incision may be needed to provide wider exposure to the upper clival region. However, temporal lobe retraction is needed, which may result in temporal lobe contusion and prolonged venous compression (2, 3, 8–10).

The retrosigmoid approach and combined transpetrosal approach were also reported to provide direct visualization of the brain stem. However, great depth, incapability to decompress the optic nerve and anterior extension of the tumor may be the limitations of these approaches (5, 6).

Recently, Bai et al. introduced an endoscopic far-lateral supracerebellar infratentorial approach for PCP meningioma removal. It provides early visualization of the oculomotor and trochlear nerves and traces them to the involved part with direct visualization of the ICA and its branches. In contrast, superior projection of the tumor might be exposed just after significant tumor removal on the inferior part. Moreover, this approach may cause injury to the venous drainage while conducting a suboccipital craniotomy (8).

### Endoscopic endonasal transposterior clinoid approach

The skull base approach focuses on several essential points, including adequate exposure to the lesion, which can facilitate resection and tumor debulking with adequate dural repair procedures. The approach must allow for the possibility of expanding the exposure if there is an extension of the tumor margin (20, 21).

In the past decade, there has been significant advancement in the development of EEA. Compared to the transcranial skull base approach, the endonasal technique provides the most direct anatomical and less invasive technique while avoiding manipulating the neurovascular structures to approach the midline skull base (8, 22, 23). This approach offers good magnification and illumination to the lesion, thus facilitating the surgeon to visualize the midline skull base region without requiring brain manipulation. These modifications have been employed for the management of several skull base pathologies. Moreover, improvements in image navigation systems utilized in endoscopic endonasal surgeries have resulted in greater accuracy and safety for this surgery. These improvements enable surgeons to maintain a consistent and improved surgical orientation inside anatomically intricate regions (12, 20, 21, 23–25).

The visualization provided by the microscope to reach the centered extra-axial skull base lesion will be obstructed by several critical neurovascular structures due to the tunnel vision possessed by the microscope, resulting in a very narrow operating corridor, thus making this approach challenging to perform tumor resection and increase the risk of injury to the structures (17, 22). Additional brain manipulations are also needed to visualize and reach the tumor location. The EEA can circumvent these limitations because it gives us a panoramic view to expose centered skull base lesions, without any brain manipulation. Furthermore, by using a variety of angled lenses, the surgeon can evaluate the anatomical border of the tumor up to the far corners (20, 22).

PCP meningioma is an extra-axial midline skull base lesion. The natural growth of the tumor in the midline area is the direction of growth from the medial to the lateral so that it laterally pushes the surrounding neurovascular structures (20). This issue is vital to select the approach as EEA provides the most direct pathway and starts from the center of the tumor in the midline area without any obstruction by surrounding neurovascular structures. Furthermore, the panoramic view allowed by EEA prevents blind manipulation, thereby reducing the risk of injuring the neurovascular structures around the tumor and ensuring gross total removal by resectioning the dura and bone attachments (22).

In this case, we are doing a posterior clinoidectomy by detaching the PCP from its surrounding bony and ligament structures. Safety drilling was implemented by drilling the bony structure around the carotid canal and clival region until the paper-thin appearance, then totally removed using Kerrison rongeur. Direct removal of the bone surrounding the ICA by Kerrison rongeur presents challenges as the bone is compound and formidable to break. The cavernous sinus and periosteal dura, which are situated medially to the ICA, have a protective function, and the bleeding from the cavernous sinus may act as a patrol of ICA exposure, which could increase the risk to injures the ICA. This technique is regarded as a more rational approach for minimizing the occurrence of bleeding and reducing the risk of harm to adjacent organs. Then, the computed tomography scan showed that the PCP is already hyperostotic due to tumor infiltration, so direct posterior clindoidectomy is considered more impractical. This technique also does not need additional pituitary transposition or any exposure of the cavernous sinus that may lead to further complications.

The dorsal meningeal artery serves as the primary source of blood supply for PCP meningioma. Its identification is easily feasible at the earliest stages of surgical intervention, facilitating the subsequent operative procedures. When the surgeon performs tumor debulking with minimum bleeding, it mitigates the requirement for the surgeon to experience exhaustion. Moreover, PCP meningioma is a tumor exclusively attached to PCP. Consequently, performing a posterior clinoidectomy at an early stage of the surgical procedure makes the tumor more mobile and less resistant to manipulation. This concept is essential as the tumor exhibits more mobility in this stage, and surgeons are allowed for a more efficient and safer tumor dissection. In this case, we use a 30-degree angled scope to visualize the base of the tumor and 70-degree angled scope to remove the superior and anterior part of the tumor around the pituitary stalk, optic tract and optic chiasm.

Despite all its advantages, we realize this approach has some limitations. Using this approach, one can encounter significant venous bleeding originating from the circular sinuses, cavernous sinuses, intercavernous sinuses, and the venous plexus in the dura mater around the clivus. This condition can be overcome by packing the venous pool using a hemostatic agent as well as cottonoid, which is challenging to do so because the working space is relatively narrow compared to transcranial access, so it requires experience from a neurosurgeon to deal with this bleeding (8, 20, 22).

Furthermore, this approach carries a higher risk of CSF leakage. It is the most common complication to be encountered with the extended endoscopic skull base approach, with an incidence ranging from 1.5 – 6.4% (20). In the EEA for skull base meningioma, we removed most of the tumor-infiltrated bone along with the ossified dura resulting in a significant defect in the skull base. Several techniques have been employed to mitigate CSF leaking in EEA procedures. In this case, the reconstruction of the skull base is accomplished by employing an analogous dural substitute alongside an abdominal fat graft positioned both within and outside the dura with dural stitches to maintain its integrity (26). Finally, Superfixorb^®^ was employed as a solid element to minimize the risk of our multilayer closure displacement.

Lastly, this approach was limited by tumor extension laterally along the petroclival junction that was hindered by the petrous apex. In this case, the translacerum is needed to expose the paraclival ICA and transpose it laterally to achieve adequate visualization of the petrous apex and drilled it out (27). The procedure involves the removal of the medial root of the pterygoid process, paraclival carotid artery and cartilaginous segment of the Eustachian tube. This technique generates a triangular-shaped space which is defined by the lower portion of the horizontal segment of the petrous carotid artery, the upper portion of the Eustachian tube (ET), and a line extended along the medial aspect of the paraclival carotid artery. Subsequently, a drilling procedure was executed along this corridor, accompanied by the ICA transposition to expand the surgical corridor.

### Patients perspective

In our case, the patient presented with a headache for the last two months, which prompted the patient to seek medical attention and planned for a brain MR imaging. The MR imaging revealed that she had a skull base tumor with brain stem compression. After discussing with the family, the patient approves our advice to undergo surgery for endoscopic endonasal tumor removal. The postoperative course was uneventful. The patient was greatly helped by the surgical procedure and appreciated the surgical team’s decision. Likewise, during the follow-up, the patient felt her complaints were improving. This condition causes the patient to feel grateful, especially since the postoperative MRI revealed gross total tumor removal.

## Conclusion

PCP meningiomas are an exceedingly rare entity. To the best of the author’s knowledge, this is the first reported case of implementing the EEA for treating PCP meningiomas. Overall, compared to transcranial approach, the EEA provides tumor removal through its natural corridor with relatively lower morbidity and provides neurosurgeons with alternative surgery for PCP meningioma cases. However, the effectiveness of this approach for PCP meningiomas requires further verification.

## Ethics statement

Written informed consent was obtained from the individual(s)/minor(s)' legal guardian/next of kin, for the publication of any potentially identifiable images or data included in this article.

## Author contributions

SA: Conceptualization, Investigation, Methodology, Project administration, Visualization, Writing – original draft, Writing – review & editing. KT: Conceptualization, Investigation, Project administration, Visualization, Writing – review & editing. EI: Writing – review & editing. YN: Writing – review & editing. NG: Supervision, Writing – review & editing. TM: Supervision, Writing – review & editing. RS: Supervision, Writing – review & editing.

## Supplementary material

Supplementary Data Sheet 1 A 52-year-old woman was brought to the outpatient clinic complaining about headaches for approximately the last two months. There is no history of seizures, nausea or vomiting. Both ophthalmological and neurological examinations are within normal limits. We are performing an endoscopic endonasal approach through a transseptal approach. We perform the right posterior clinoidectomy by drilling dorsum sella, deskeletonizing the right carotid canal and disconnect the posterior clinoid with the posterior petroclinoidal ligament. After that, we visualize the main feeder of the tumor by the dorsal meningeal artery, coagulate and cut it to devascularize the tumor. Linear dural incision was made to visualize the tumor. Tumor debulking was done by using tumor forceps along with a surgical aspirator. Detachment of the tumor from the surrounding structure is safely performed by mobilizing the tumor. After removing the tumor, we evaluated the surrounding neurovascular structure. Multilayer dural closure was used using an inlay and onlay fashion along with absorbable bone, the sphenoid mucosal flap and fibrin glue.
